# Non-Instrumental Movement Inhibition (NIMI) Differentially Suppresses Head and Thigh Movements during Screenic Engagement: Dependence on Interaction

**DOI:** 10.3389/fpsyg.2016.00157

**Published:** 2016-02-23

**Authors:** Harry J. Witchel, Carlos P. Santos, James K. Ackah, Carina E. I. Westling, Nachiappan Chockalingam

**Affiliations:** ^1^Brighton and Sussex Medical School, University of SussexBrighton, UK; ^2^School of Media Film and Music, University of SussexBrighton, UK; ^3^Centre for Sport, Health and Exercise Research, Staffordshire UniversityStoke-on-Trent, UK

**Keywords:** posture, movement, human, cognitive engagement, NIMI, motion capture, video tracking, non-instrumental movement inhibition

## Abstract

**Background:** Estimating engagement levels from postural micromovements has been summarized by some researchers as: increased proximity to the screen is a marker for engagement, while increased postural movement is a signal for disengagement or negative affect. However, these findings are inconclusive: the movement hypothesis challenges other findings of dyadic interaction in humans, and experiments on the positional hypothesis diverge from it.

**Hypotheses:** (1) Under controlled conditions, adding a relevant visual stimulus to an auditory stimulus will preferentially result in Non-Instrumental Movement Inhibition (NIMI) of the head. (2) When instrumental movements are eliminated and computer-interaction rate is held constant, for two identically-structured stimuli, cognitive engagement (i.e., interest) will result in measurable NIMI of the body generally.

**Methods:** Twenty-seven healthy participants were seated in front of a computer monitor and speakers. Discrete 3-min stimuli were presented with interactions mediated via a handheld trackball without any keyboard, to minimize instrumental movements of the participant's body. Music videos and audio-only music were used to test hypothesis (1). Time-sensitive, highly interactive stimuli were used to test hypothesis (2). Subjective responses were assessed via visual analog scales. The computer users' movements were quantified using video motion tracking from the lateral aspect. Repeated measures ANOVAs with Tukey *post hoc* comparisons were performed.

**Results:** For two equivalently-engaging music videos, eliminating the visual content elicited significantly increased non-instrumental movements of the head (while also decreasing subjective engagement); a highly engaging user-selected piece of favorite music led to further increased non-instrumental movement. For two comparable reading tasks, the more engaging reading significantly inhibited (42%) movement of the head and thigh; however, when a highly engaging video game was compared to the boring reading, even though the reading task and the game had similar levels of interaction (trackball clicks), only thigh movement was significantly inhibited, not head movement.

**Conclusions:** NIMI can be elicited by adding a relevant visual accompaniment to an audio-only stimulus or by making a stimulus cognitively engaging. However, these results presume that all other factors are held constant, because total movement rates can be affected by cognitive engagement, instrumental movements, visual requirements, and the time-sensitivity of the stimulus.

## Introduction

### Practical uses of measuring non-instrumental postural movements

Instrumental movements are fundamental to the process of the task at hand; for a person interacting with a computer, they include using the mouse (or any controller such as a keyboard), postural actions required to use the controller (e.g., leaning forward), and head and eye movements that are used for targeting gaze. Non-instrumental movements are not task-required, e.g., fidgeting, scratching, stretching, and emotional expressions; although not task-required, they are often unwittingly task-induced via cognitive states (Ekman and Friesen, 1969; Mehrabian, 1969).

The rationales for linking assessments of nonverbal behavior (such as task-induced non-instrumental movements and gestures) to cognitive states are two-fold: (1) there is a long-standing scientific literature on nonverbal behavior and its meaning (Bull, 1987). (2) The engineering and applied behavior literature seeks to recognize human cognitive states and emotions via various nonverbal behaviors.

Recognition in this way will be important in responsive learning systems such as automated tutors (D'Mello et al., 2007), virtual humans seeking to achieve rapport with patients (Gratch et al., 2014), companion robots and service robots (Huth and de Ruiter, 2012). For example, in automated tutoring systems, the responsive system will be able to detect boredom or frustration of the learners before they disengage.

Postural movements, in particular, including movements of the head (D'Mello et al., 2012), torso (Grafsgaard et al., 2012), and hands (Grafsgaard et al., 2013) and how these relate to engagement and other cognitive states, are extensively researched in the engineering and human-computer interaction (HCI) literature as potential metrics to obtain continuous, objective data/information on engagement. Currently these movement analyses have a fundamental problem in that there is no easy way for an automated system to distinguish instrumental from non-instrumental movements.

### Position (approach-withdrawal) vs. net movement as markers of engagement

Some folk psychology theories (Pease and Pease, 2004; Sandberg, 2013), and many scientific studies on interpreting nonverbal behavior (James, 1932; Coan and Gottman, 2007; Rodrigo and Baker, 2011; Sanghvi et al., 2011), have suggested that leaning forward (i.e., seated approach) is a postural marker of engagement. When simple averages of head distance-to-screen are made, this proposal is not supported (Mota and Picard, 2003; Witchel et al., 2013a,b, 2014a). Our group and others have pointed out that forward-leaning load-bearing postures, where the head rests on the hand(s), are usually associated with boredom, disengagement, or difficulty, despite the fact that these postures are invariably associated with more forward leaning than most other seated postures (Grafsgaard et al., 2013; Witchel et al., 2014a). The use of position as a marker of engagement remains controversial except when detecting outright sleep (e.g., during night driving).

Our team recently demonstrated that Non-Instrumental Movement Inhibition (NIMI) could manifest as a marker for cognitive engagement in seated computer users (Witchel et al., 2014a). Likewise, other research has identified many behaviors (smiling, talking, making sounds, head movements) that are inhibited during interaction with intelligent (i.e., computer-based) tutors in the classroom when students are “on task” (i.e., during engagement; Woolf et al., 2009).

In the psychology literature, many gestures and non-instrumental movements during dyadic communication have been associated with increasing engagement; for example, a sitting speaker is over 10 times more likely to draw their legs backward when speaking on an interesting topic than when bored (Bull, 1987). Just listening during conversation is sufficient to cause listeners to make a range of movements including backchanneling (McClave, 2000; Kogure, 2007). Another listener movement associated with engagement is unintentional interactional synchrony, when subconscious movements by the speaker and listener co-occur with synchronized timings (Condon, 1980; Schmidt et al., 2012). Acts of interactional synchrony are fundamental to engagement during dyadic interaction; for example, these movements are correlated with the quality of infant-mother attachment (Isabella and Belsky, 1991), successful psychotherapy (Ramseyer and Tschacher, 2011), increased motivation for pro-social behaviors (Van Baaren et al., 2004), and successful dyadic negotiations (Park et al., 2013). Even when a speaker is not present, such as when the listener is seated and watching a movie of a lecture, arousal associated with interest can result in an increase of some postural movements such as leaning forward (Bull, 1987).

There is tacit disagreement in HCI over the interpretation of increased levels of movement. Bianchi-Berthouze and colleagues, testing standing game players, showed that task-related movement results in greater subjective engagement (Bianchi-Berthouze et al., 2007), and that head movements could be used to discriminate between low and high levels of arousal, valence and potency. Mota and Picard (2003) testing seated 8–11-year-old children playing the game Fripple Place measured postural movement with a pressure sensitive chair mat, and showed (using ground truth engagement agreed by children's teachers) that leaning forward repeatedly was a measure of engagement. However, other HCI researchers, making body motion measurements on seated volunteers viewing media or interacting with screens, have concluded that increased movements are associated with either diminished engagement or negative emotions.

For example, Kapoor et al. (2007) found that head velocity was a reliable indicator of self-identified frustration in 12–13-year-old children working on a computer version of the Towers of Hanoi puzzle. D'Mello et al. (2007) included increased change rate in seat pressure as an indicator of boredom during a physics learning session with an automated tutor system. Grafsgaard et al. (2012) testing a computer-mediated human-human tutoring system teaching Java to university students found that diminished head movement was related to engagement, and that increased overall body movement was related to frustration. Woolf et al. (2009) tested children in a classroom using a mathematics intelligent tutor, and found that high levels of head movement were correlated with negative valence, high arousal, off-task behavior, and non-desirable states. One HCI group recently made the broad claim, that “increases in postural movement are linked with negative affect or disengagement” (Grafsgaard et al., 2013).

This claim relies on being able to distinguish instrumental from non-instrumental movement; for example, one can minimize instrumental movements using a handheld trackball (which only requires finger activity, and does not interfere with free arm or shoulder movement) to eliminate instrumental movements except those associated with gaze and with the fingers. However, continuous stimuli used to compare engagement to disengagement do not tend to control for interaction rate or instrumental movement in their analyses of movement, and thus previous studies have not differentiated instrumental movements from non-instrumental movements. Thus, in video games or intelligent tutor examples (Mota and Picard, 2003; D'Mello et al., 2007; Kapoor et al., 2007; Woolf et al., 2009; Rodrigo and Baker, 2011; Grafsgaard et al., 2012), lower engagement would be associated with lower interaction rates. However, low interaction rates when quietly engaged can resemble boredom, while high interaction rates when dynamically engaged can create entrained activity levels that read as frustration when measured automatically (Witchel et al., 2014b). For example, when comparing three customized levels of a first person shooter video game meant to induce boredom (easy level), enjoyment (moderate level), and frustration (hard level), the version associated with the most measurable activity is the frustrating level (as predicted by claims about non-instrumental behavior). However, that level was also the most difficult, and thus, the increased upper body movement rate could have reflected either induced frustration (non-instrumental behavior) or physical engagement due to increased demands leading to an increased interaction rate (instrumental activity; van den Hoogen et al., 2008). In part, these confusions arise due to the conflation of cognitive engagement with physical engagement.

### Engagement vs. attention

Engagement and attention are related but not equivalent. Attention can mediate instantaneously between many competing stimuli, while engagement lasts longer, is not as completely exclusive, and implies at least a partial commitment to action or volition (Henrie et al., 2015). As such engagement falls within the remit of applied psychologies including work psychology (Kahn, 1990; Macey and Schneider, 2008; Christian et al., 2011), educational psychology (Finn and Zimmer, 2012), positive psychology (Csikszentmihalyi, 1998), and human computer interaction (Webster and Ho, 1997; O'Brien and Toms, 2010). It is possible to have attention without engagement, and engagement without attention. Driving a car while day-dreaming is an example of attention with minimal engagement, while introspecting on the implications of a lecture you are currently attending is an example of high engagement without paying second-by-second attention.

Because engagement is longer in duration and not completely exclusive, it can be defined as a family of related cognitive states geared toward extended interaction and/or a purposeful outcome, operationalized by a collection of behaviors, none of which are absolutely necessary at a given point in time, including: attendance, attention, memory, caring, emotion, taking action, making an effort, and (like the exclusion in attention) inhibition of irrelevant activities (Witchel, 2013). For example, during a lecture, playing video games, or private conversations are irrelevant activities that are inhibited during student engagement.

Most commentators on engagement subdivide its manifestations into categories such as cognitive, emotional/affective, and physical/behavioral/motor engagement (Bloom, 1956; Kahn, 1990; Macey and Schneider, 2008). The NIMI concept allows for a comprehensive definition of engagement by mediating between these different categories of engagement. In seated individuals, rapt engagement (e.g., with a film) has an exclusory role, in that the cognitive engagement manifests as an inhibition in physical activity (Witchel et al., 2014a); put another way, this physical engagement with the screen suppresses other actions that might otherwise be interpreted as physical engagement. Confusion in the literature arises because some technology-keen educators have effectively equated cognitive engagement with increased interactivity manifesting as physical activity (Northrup, 2001), yet, in some cases (e.g., seated, “passive” screenic engagement) increasing engagement is associated with reduced physical activity, rather than with more physical activity.

### Head posture is limited during cognitive screenic engagement

The total movements of any body part is a mixture of instrumental and non-instrumental movements, and the non-instrumental movements of the head will be limited by the instrumental needs of where a person needs to look. For example, a person watching a television has to face the screen in order to fully engage with the visual content, while a person listening to the radio can face in any direction and still listen to (and fully engage with) the radio content. Hypothetically, targeting the gaze to watch something on a fixed-position screen should concurrently suppress some non-instrumental head positions and movements (i.e., it elicits NIMI) compared to behaviors elicited by comparable audio-only content.

In addition to position, many body parts' velocity and speed will be affected by engagement; for example, reading text is quite difficult when nodding one's head. By contrast, thigh movements are not intimately connected to gaze. One can easily read a book while seated even when the legs are in constant motion (e.g., jigging the leg). Thus, thigh movement is not of necessity instrumentally restricted by the process of reading or screen watching.

### Stimulus duration repercussions when testing for engagement

Testing for the relationship between stimulus-elicited emotions and their manifestations is more relevant for applications when using continuous stimuli, but it should be more experimentally tractable when using short, discrete stimuli because the elicited emotions will be more predictably homogeneous. In continuous stimuli, such as long video game playing sessions, it is assumed that the end-user's emotional state varies despite the stimulus being relatively uniform (i.e., due to boredom or fatigue). In experiments using continuous stimuli, ground truth for emotions is provided either by (a) interrupting play to sample emotions, (b) asking the end-user to review films of himself/herself, or (c) asking experts to interpret films of the end-user's body activity. These techniques either assume that the player has clear insight into their own emotions, or that the nonverbal behavior interpretations of experts are true. Since part of the goal of this study was to test the current assumptions of nonverbal behavior experts, we settled on using discrete stimuli.

For brief stimuli (e.g., using the International Affective Photographic System; Lang and Bradley, 2007), 6-s stimulus-times provide a short enough time window that the emotional response can be assumed to be homogeneous. However, using 6-s intervals is inappropriate for measuring cognitive states like engagement or boredom. While there are many potential discrete stimuli for eliciting cognitive states such as boredom or engagement, in the following pair of studies we designed special stimuli that were made to be as comparable to each other as possible, in order to make clear, controlled tests on the relationship between cognitive states (especially cognitive engagement) and elicited non-instrumental movements during non-brief (i.e., over 1 min) activities. That is, this work attempts to bridge the gap between measuring attention and measuring engagement.

## Hypotheses

In study 1, we sought conclusive data to support the hypothesis that adding visual content to an audio-only stimulus will decrease head movements (but not necessarily thigh movements), irrespective of whether the subjective engagement in the multimodal stimulus is modestly higher or lower than during the audio stimulus. That is, so long as the viewer makes an effort to attend to the screen (whether or not they feel the content is engaging), they will suppress large non-instrumental movements that might cause their head to face away from the screen; by contrast, audio-only stimuli (even when engaging) allow for large non-instrumental head movements. This experiment will support the idea that attention and targeting gaze has an inhibitory effect on non-instrumental head movements.

In study 2, we designed stimuli to test directly whether cognitive (rather than physical) engagement itself is responsible for lowering non-instrumental movement in the body generally, independent of interaction rate. We created special reading comprehension test stimuli with high interaction rates (27 clicks per min), while instrumental movements were minimized by mediating all interactions via a handheld trackball. Interest should be sufficient for reducing head and postural movements, which we term NIMI. Also, in both studies we tested the hypothesis that engagement is associated with the seated computer user approaching the screen, with measurements of mean head distance from the screen.

Both studies were organized using a single independent variable with three levels; the independent variable being tested was the stimulus. The rationale for using three levels to test one hypothesis was to support the hypothesis with two comparable stimuli, but also for the third stimulus to provide an exception to the oversimplified hypothesis that cognitive engagement always diminishes total movement. In both studies the third stimulus was highly engaging while varying the visual demands upon the viewer, in order to demonstrate the dangers of conflating non-instrumental movement with total movement. Thus, the third stimulus in each study demonstrates the need for the NIMI concept to avoid this conflation, by exemplifying the apparent exceptions to the oversimplified hypothesis.

## Study 1: Multimodal stimuli elicit NIMI with respect to comparable audio-only stimuli

There are several theoretical causes for a seated person interacting with a computer to move less: targeting gaze and attention, rapt engagement, increased mouse/keyboard interactions (or other instrumental actions that lock the shoulder in place), and lethargic boredom. As a precursor to testing the effect of cognitive engagement on movement in study 2, we verified in study 1, that attractive and relevant on-screen visual stimuli can lead to diminished movement. To test this hypothesis, we used two passive (i.e., non-interactive) stimuli that were similar in terms of audio. It may seem self-evident that having something to look at will cause the head to move less, but this implies that being engaging *per se* is not sufficient to reduce head movements. Furthermore, it was not clear whether the visual stimulus would inhibit thigh movement as well as head movement.

### Methods

#### Sample and study design

Twenty-seven healthy participants (15 women, 12 men; mean age 21.00, *SD* = 2.89) were tested. This study was carried out in accordance with the approval of BSMS's Research Governance and Ethics Committee (RGEC), with written informed consent obtained from all subjects. All subjects gave written informed consent in accordance with the Declaration of Helsinki. Participants, mostly students, were recruited via an email to the university community and received £20 for their travel and/or time.

The single independent variable was stimulus, of which there were three: FAV (having no visual component), OK Go video (multimodal), and OK Go audio-only. The purposeful planned comparison (video vs. audio-only for the OK Go songs) was meant to be accompanied by the important exception: that highly engaging audio-only FAV was expected to elicit more head movement than less engaging video stimuli. The primary dependent variables were subjective response (Visual Analog Scale) for “I felt totally engaged,” and the speed of movement of the reflective motion capture markers on the body.

We have designed these experiments to be repeated measures comparisons, despite the fact that two different songs by OK Go were used in each of the conditions (video vs. audio-only). There is a precedent for grouping different pieces of music as a single type of stimulus; when scientists need to elicit strong responses to music, they ask for user-selected favorite music and analyze it as a group (Blood and Zatorre, 2001). Blood and Zatorre went so far as to use the favorite music of other volunteers as control music (i.e., the favorite music of volunteer A was the control music for volunteer B).

When considering what drives the responses to different songs, there are four relevant factors: the song itself, the medium (video vs. audio-only), consistent issues relating to the individual participant, and an error factor. The design of a repeated measures analysis relates to the planned consistencies in participant factors, which are quite strong in our experiments. For example, in subjective measures there are consistencies within individual participant measurements, such as how interested they are in taking part in a psychological experiment (which would increase ratings of engagement for most stimuli). Likewise, how fidgety a participant is can affect all their head movements.

#### Instruments and scales

After each 3-min stimulus, participants completed both the Self-Assessment Manikin (SAM) and a collection of Visual Analog Scales (VAS); these are 10 cm rating scales with anchors at 0 (“Not At All”) and at 100 (“Extremely”). The scales were “I wanted to see/hear more,” “I felt totally engaged,” “I felt interested.” “I wanted it to end earlier,” “I felt bored,” “I felt frustrated.” In this study, we have operationalized engagement by asking the participants to subjectively assess their own engagement.

The SAM (Bradley and Lang, 1994) asks for three user ratings of Quiet-to-Active (i.e., arousal), Sad-to-Cheerful (valence), and Independence-to-Dependence (dominance), to reproduce a 3-dimensional range of emotional experiences, such as in (Russell and Mehrabian, 1977; Mehrabian, 1996).

#### Procedure

The methodology of this kind of study has been described previously (Witchel et al., 2013b), although the stimuli used for this experiment were new. Participants were initially briefed about the experiment, although the precise role of the camera and postural movement was not explained until the de-briefing. Consent was then agreed in writing. Motion capture markers were affixed to skin or clothing on the side of the body facing the camera on: the forehead, the pinna of the ear, the badge of the deltoid, the greater trochanter, the mid-thigh, and the proximal end of the fibula. Electrodes for the ECG were fitted on the shoulders (data to be reported later), and the participant received instructions on filling in SAM and the VAS. The participant completed questionnaires including a demographics questionnaire and the Berkeley Expressivity Questionnaire.

**Figure 1:**
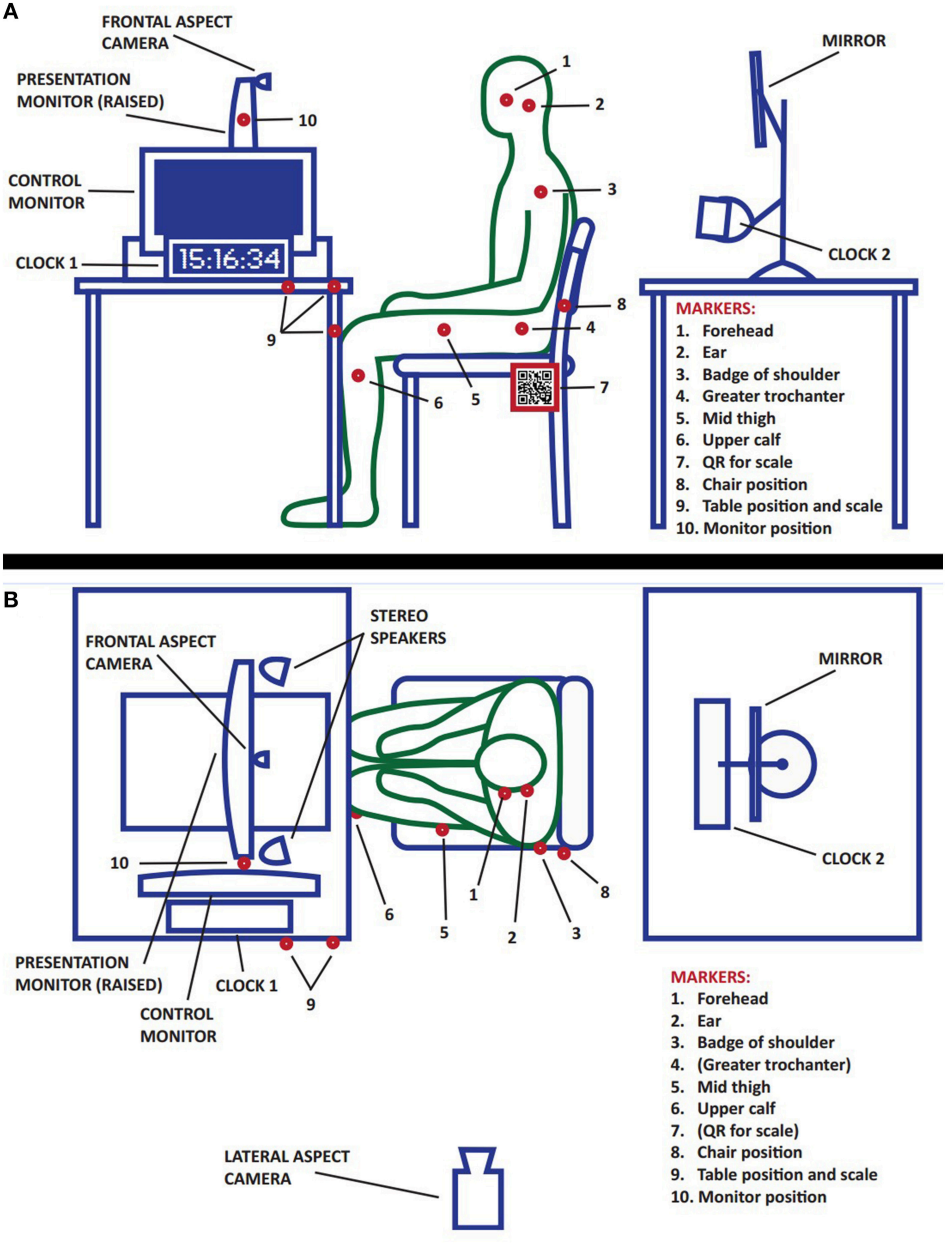
**Experimental set up: computer, participant and camera**. In panel **(A)**, the experimental set up is shown side-on from the left (as if looking from the point of view of the lateral camera). Red dots represent the placement of reflective motion capture markers that are tracked by our system. Note that the stereo speakers are not visible because they are behind the stimulus control laptop. Clock 2 and the mirror are for use with the frontal aspect camera (focused on the face, data not presented in this study). In panel **(B)**, the set up is seen from above. The motion capture marker balls for the greater trochanter and the QR code are not visible from this aspect.

The experimental set up is shown in Figure 1. The stimulus laptop faced away from the participants, and a second monitor facing the participant had its height adjusted so that the center of the monitor was at eye height of the participant, so that the head's pitching forward due to fatigue could be distinguished from gazing (down) toward the screen. The mouse interactions were conducted with a handheld trackball, which minimized the need for instrumental hand movements, as well as eliminating the limitations of shoulder movement associated with mouse use. Participants were invited to move the armless “reception-style” chair into a comfortable position for seeing the monitor and for using the trackball during game-play. Participants were also invited to adjust the volume of the portable speakers using a knob on the speakers. Two initial stimuli (for training and habituation) were always used, followed by a counterbalanced order of the experimental stimuli. Beforehand, if the stimulus was interactive, participants were given the trackball to hold in any way they preferred. During each stimulus the experimenters were outside the room, and at the end of 175 s the volunteer was asked to complete the questionnaire. After all stimuli were completed, markers and electrodes were removed, and participants were de-briefed and paid. Participants experienced all stimuli for both studies (total participation lasting under 60 min) to allow direct comparisons between the movement data.

#### Data analysis

Digital films of body movement were captured as 3 min fragments for each stimulus using Movie Maker on computers running Windows. The motions of all reflective markers in two dimensions were tracked using Kinovea 1.0, and two-dimensional known length standards (10 cm QR codes that can be recognized by computer) were used to calibrate the Kinovea measurements. These two-dimensional measurements of movements were previously shown to be highly correlated with three-dimensional movements captured with the gold standard (Vicon opto-electronic motion capture system; Witchel et al., 2012). Matlab was used to translate the xml data files exported from Kinovea into Matlab structures, to calibrate and then parse the data into blocks for analysis.

The parsing of each time course into an 82-s segment is described in full in (Witchel et al., 2013b). In brief, for each stimulus the algorithm selects 82 continuous seconds of activity that ends 13 s before the end of each 175-s stimulus; this selection allows for the participant to settle in to each homogeneous stimulus, while avoiding transition periods that might enhance activity. Time courses for the x,y position (i.e., sagittal plane) at 25 Hz were low pass filtered and the absolute value for Euclidean distance between adjacent time points (divided by the inter-sample interval) was calculated to determine the speed. The two features used in this study were position and speed, each based upon mean values for the entire time course selections lasting 82 s. We chose these long time periods to assess engagement in order to maximize the movements we detected, because we need to detect occasional movements in order to measure inhibition of movements (i.e., NIMI). This fits with our model of boredom being lethargy punctuated by occasional, brief periods of restlessness (Witchel et al., 2014b).

Statistical analyses of subjective and movement data were performed in Matlab. ${\hat{\omega }}^{2}$ was chosen as a measure of unbiased effect size for ANOVAs (Snyder and Lawson, 1993), and calculated according to the method of Olejnik and Algina (2003).

#### Stimuli

Passive stimuli were audiovisual stimuli without the requirement for interaction (e.g., mouse activity). Each stimulus lasted 175 s, with source videos being cut-short to fit our format. Two similarly engaging music videos by the band OK Go (Supplementary Figure 1) were duplicated by the experimenters, one copy of each had the video content removed (i.e., the computer user was listening to music in front of a black screen). A third musical piece, FAV, was a favorite upbeat piece of music selected by the participant beforehand. In summary, during study 1 each volunteer experienced three musical stimuli: OK Go audio-only, OK Go video (multimodal), and FAV, in a counterbalanced order (see Supplementary Table 1).

One experimental design issue is that for a comparison of a music video with and without the video in a paired design, the same song could not be experienced twice because the second music video stimulus would be subject to habituation and boredom. To avoid this we used two popular videos made by the same band (OK Go) that elicited very similar levels of subjective engagement (see Tables 1, 2). The music videos of OK Go were selected because they are very popular, action-packed, and visually arresting; among the age-group of our participants, the music of OK Go is generally viewed positively, and rarely viewed negatively. In total, there were four OK Go music video stimuli but each volunteer only experienced two of them. The two training stimuli were reported on previously (Witchel et al., 2013b).

**Table 1:** **Summary of the musical stimuli used in this experiment**.

**Stimulus**	**Modality**	**Expected design goal**
**MUSIC VIDEOS BY OK GO**		
Song 1: Here It Goes Again (HIGA)	Multimodal	Moderately engaging
Song 1: Here It Goes Again (HIGA)	Audio only	Partially engaging
Song 2: Do What You Want (DWYW)	Multimodal	Moderately engaging
Song 2: Do What You Want (DWYW)	Audio only	Partially engaging
**USER-SELECTED MUSIC**		
Music: FAV	Audio only	Very engaging

*Modality refers to whether the stimulus is presented via vision, sound or both. The multimodal condition refers to the participant watching one of two truncated music videos by the band OK Go: “Here It Goes Again” (Treadmills) or “Do What You Want” (Wallpaper version). The audio only condition refers to the same OK Go music videos in which the video has been replaced by a black screen (i.e., no video). FAV, a favorite song self-selected by the participant in advance and played for 175 s during the experiment (i.e., the computer monitor screen was black). Expected design goal refers to the a priori subjective response the research team wanted to elicit from the participants*.

**Table 2:** **Mean subjective rating elicited by each musical stimulus, and by each category of musical stimulus**.

	**Study 1**
	**Engaging** *M(SD)*
**MUSIC BY THE BAND OK GO**	
Audio only: Pooled OK Go	32.78 (21.05)
Audio only: Here It Goes Again	34.64 (15.99)^†^
Audio only: Do What You Want	30.77 (25.97)^*^
Multimodal: Pooled OK Go	61.11 (17.83)
Multimodal: Here It Goes Again	60.38 (19.41)^*^
Multimodal: Do What You Want	61.79 (16.94)^†^
**MUSIC SELF-SELECTED BY PARTICIPANTS**	
Audio Only: FAV (a favorite piece)	71.85 (20.62)

Ratings were made immediately after the stimulus on a 0–100 Visual Analog Scale. Engaging, “I felt totally engaged by the experience.” The multimodal condition refers to the participant watching one of two truncated music videos by the band OK Go: “Here It Goes Again” (Treadmills) or “Do What You Want” (Wallpaper version). The audio only condition refers to the same OK Go music videos, but where the video content has been replaced by a black screen (i.e., no video). Pooled conditions are where the statistical analysis considers the rating for either song (all participants experienced only one of the two conditions for each song). FAV, a favorite song self-selected by the participant in advance and played for 175 s during the experiment (i.e., the computer monitor screen was black). The N numbers depend on the particular stimulus, but for pooled stimuli N = 27, for individual stimuli ^*^N = 13, ^†^N = 14.

### Results: Passive musical stimuli

#### Stimuli comparability

As expected, there were no significant differences in engagement between the two multimodal stimuli (i.e., as music videos) or the two audio-only stimuli (i.e., as songs, with the computer screen being completely black); that is, the songs Here It Goes Again (HIGA) and Do What You Want (DWYW) did not differ in engagement (see Table 3), except when presented in a different modality. Thus, the two OK Go songs (and their music videos) were comparable in terms of their engagement (see Table 3), interactivity rates, and visual requirements, and all further analyses have pooled the two different OK Go songs, in order to address the main hypothesis (using paired statistics in a within-subjects design) as to how much the effects of these music videos differed if they were presented multimodally or in an audio-only format. The participant's favorite song could, according to some hypotheses (Grafsgaard et al., 2013), lead to less movement because it would be expected to be more engaging and less frustrating.

**Table 3:** **Comparison of subjective responses elicited by each musical stimulus derived from OK Go music videos**.

**Stimulus 1**		**Stimulus 2**					
**Modality**	**Song**	**Modality**	**Song**	**95% CI Lower bound**	**Difference of Means (1–2)**	**95% CI Upper bound**	***P***
**TOTALLY ENGAGED (***N* = 27**)**							
Multimodal	HIGA	Multimodal	DWYW	–20.94	–1.41	18.38	0.9981
Audio only	HIGA	Audio only	DWYW	–16.76	3.88	22.56	0.9795
Multimodal	HIGA	Audio only	HIGA	7.06	25.73	46.38	0.0038
Multimodal	DWYW	Audio only	DWYW	11.24	31.02	50.56	<0.001

*These are post-hoc statistical comparisons (Tukey) of a repeated measures ANOVA. If the difference of means (1–2) is positive, then the mean Visual Analog Scale subjective response elicited by stimulus 1 was higher than stimulus 2, while stimulus 2 elicited a higher mean subjective rating if the difference is negative. HIGA, Here It Goes Again (Treadmills). DWYW, Do What You Want (Wallpaper version). See Methods for details*.

#### Subjective ratings

Subjectively, the outright loss of visual content in the matched music videos made the stimulus less engaging; the subjective responses for VAS engaged were significantly different between multimodal vs. audio-only stimuli [see Table 2, Repeated Measures ANOVA, *p* < 0.001, *F*(2, 80) = 36.6, ${\hat{\omega }}^{2}$ = 0.56, *post-hoc* Tukey comparison, see *post-hoc* comparisons Table 4]. As expected, the participants' ratings for their self-selected favorite songs (FAV) were highly significantly more engaging than the audio-only OK Go musical excerpts. Furthermore, despite the fact that the favorite songs were accompanied by a black screen, they showed a trend for being more engaging than the multimodal music videos (*p* = 0.068). Ratings of frustration appeared as the following order (starting with most frustrating): audio only = multimodal > FAV [see Table 2, ANOVA, *p* < 0.001, *F*(2, 80) = 9.16, ${\hat{\omega }}^{2}$ = 0.23, *post-hoc* Tukey comparison, see *post-hoc* comparisons Table 4]; thus, in the carefully matched OK Go condition, the presence or absence of video did not significantly change the ratings of frustration, and FAV was significantly less frustrating than either OK Go stimulus.

**Table 4:** **Comparison of subjective responses elicited by each category of musical stimulus**.

**Stimulus 1**	**Stimulus 2**	**95% CI Lower bound**	**Difference of Means (1–2)**	**95% CI Upper bound**	***P***
**TOTALLY ENGAGED (***N* = 27**)**					
Audio only	Multimodal	–39.72	–28.33	–16.95	<0.001
Music: FAV	Audio only	27.69	39.07	50.46	<0.001
Music: FAV	Multimodal	–0.64	10.74	22.12	0.0682
**FRUSTRATED (***N* = 27**)**					
Audio only	Multimodal	–3.81	6.19	16.18	0.3023
Music: FAV	Audio only	–27.47	–17.48	–7.49	<0.001
Music: FAV	Multimodal	–21.29	–11.30	–1.31	0.0232

These are post-hoc statistical comparisons (Tukey) of a repeated measures ANOVA. If the difference of means (1–2) is positive, then the mean Visual Analog Scale subjective response elicited by stimulus 1 was higher than stimulus 2, while stimulus 2 elicited a higher mean subjective rating if the difference is negative. The multimodal condition refers to the participant watching one of two music videos by the band OK Go: “Here It Goes Again” (Treadmills) or “Do What You Want” (Wallpaper version). The audio only condition refers to the same OK Go music videos in which the video has been replaced by a black screen (i.e., no video). FAV, a favorite song self-selected by the participant in advance and played for 175 s during the experiment (i.e., the computer monitor screen was black). Frustrated, “I felt frustrated by the experience.” Totally engaged, “I felt totally engaged by the experience.”

#### Head movements

Listening to preferred music, can elicit a range of non-instrumental movements in the listener, often at a subconscious level (Witchel, 2011). If the music has a strong beat, the listener may entrain in various ways with the music (e.g., toe tapping, finger tapping, or even head-nodding, see Supplementary Figure 2 and Supplementary Video 1).

The three musical stimuli (FAV, audio-only, multimodal) differed highly significantly in terms of elicited head movement [see Table 5, Repeated Measures ANOVA, *p* < 0.001, *F*(2, 80) = 17.52, ${\hat{\omega }}^{2}$ = 0.38, *post-hoc* Tukey comparison, see *post-hoc* comparisons Table 6]. The multimodal stimuli (music videos) were, as expected, associated with significantly lower levels of movement of the head, compared to either of the audio-only stimuli (OK Go audio-only or FAV, see Table 5, and *post-hoc* comparisons Table 6). By contrast, the head movements elicited by either of the two audio-only stimuli (FAV and OK Go audio-only) were not significantly different from each other (Table 5).

**Table 5:** **Mean speed of movement elicited by each category of musical stimulus**.

	**Study 1 (*****N*** = 27**)**	
	**Forehead (mm/s) *M* (*SD*)**	**Thigh (mm/s) *M* (*SD*)**
Multimodal	0.29 (0.31)	0.06 (0.06)
Audio only	0.88 (0.88)	0.14 (0.12)
Music: FAV	1.09 (0.92)	0.23 (0.35)

*Speed is the absolute value of the Euclidean distance traveled in two-dimensions of a motion capture reflective marker between two samples (25 Hz); mean speed refers to the average speed over the course of 82 s (see Methods for details). The multimodal condition refers to the participant watching one of two music videos by the band OK Go: “Here It Goes Again” (Treadmills) or “Do What You Want” (Wallpaper version). The audio only condition refers to the same OK Go music videos, but where the video has been replaced by a black screen (i.e., no video). FAV, a favorite song self-selected by the participant in advance and played for 175 s during the experiment (i.e., the computer monitor screen was black)*.

**Table 6:** **Comparison of mean speed of movement elicited by each category of musical stimulus**.

**Stimulus 1**	**Stimulus 2**	**95% CI Lower bound**	**Difference of Means (1–2)**	**95% CI Upper bound**	***P***
**FOREHEAD SPEED (mm/s**, *N* = 27**)**					
Audio only	Multimodal	0.26	0.60	0.94	<0.001
Music: FAV	Multimodal	0.46	0.80	1.14	<0.001
Music: FAV	Audio only	–0.13	0.20	0.54	0.3199
**THIGH SPEED (mm/s**, *N* = 27**)**					
Audio only	Multimodal	–0.06	0.08	0.22	0.3722
Music: FAV	Multimodal	0.03	0.17	0.31	0.0139
Music: FAV	Audio only	–0.05	0.09	0.23	0.2662

*These are post-hoc statistical comparisons (Tukey) of a repeated measures ANOVA. If the difference of means (1–2) is positive, then the mean speed of movement elicited by stimulus 1 was higher than stimulus 2, while stimulus 2 elicited more movement if the difference is negative. The multimodal condition refers to the participant watching one of two truncated music videos by the band OK Go: “Here It Goes Again” (Treadmills) or “Do What You Want” (Wallpaper version). The audio only condition refers to the same OK Go music videos, but where the video has been replaced by a black screen (i.e., no video). FAV, a favorite song self-selected by the participant in advance and played for 175 s during the experiment (i.e., the computer monitor screen was black)*.

#### Thigh movements

Thigh movements differed significantly between FAV and the multimodal stimulus, but not between the two OK Go stimuli [audio-only and multimodal, see Table 5, Repeated Measures ANOVA, *p* = 0.019, *F*(2, 77) = 4.30, ${\hat{\omega }}^{2}$ = 0.11, *post-hoc* Tukey comparison, see *post-hoc* comparisons Table 6]. We conclude that visual attention due to the presence of an on-screen stimulus preferentially inhibited head movements compared to movements of the thigh.

#### Head position: Distance from monitor

In terms of mean head distance from the screen (i.e., position, rather than movement), there was a trend (paired *t*-test, *p* = 0.07, *N* = 27) for the head marker to be closer to the screen (by 7 mm) during the audio only stimulus [i.e., during the less engaging stimulus) compared to the multimodal version of the song (mean forehead distance in cm from screen (s.d.), audio-only: 73.863 (9.469); multimodal: 74.571 (9.275)]. This contradicts the expectation that increased engagement is associated with closer head position to the screen.

#### Discussion: Passive musical stimuli

This study supports the hypothesis that visual stimuli reduce head movement, and it provides an exception to the hypothesis that engagement reduces total movement. As expected, when adding appropriate video content to the OK Go songs, the resulting stimuli were more engaging while reducing head movement. This reduction in movement could be due to either needing to gaze at the monitor or to increased engagement. FAV (another audio-only stimulus) also elicited much more head movement than the multimodal stimulus. This result for FAV plainly violates the heuristic that, when seated, increased total movement implies lower engagement.

The two audio-only stimuli (FAV and audio-only OK Go) elicit much more movement than the multimodal OK Go; of the two audio-only stimuli, FAV is more subjectively engaging than the multimodal OK Go video, while the audio-only OK Go song is less engaging. This implies that, for these examples, engagement is less important in determining the amount of elicited movement than whether there is visual accompaniment, and potentially how persistently, the viewer needs to watch this, depending on whether the visual content is challenging, demanding or time-sensitive.

The difficulty of relating subjective engagement ratings to movement during non-visual musical stimuli is highlighted by our previous data showing that highly disengaging music elicits even more non-instrumental movement than favorite music in healthy male volunteers (Witchel et al., 2013a); this movement may be elicited by frustration or suppressed escape behavior, rather than by engagement.

## Study 2: Cognitive engagement elicits NIMI of both the head and the thigh during a highly interactive task

The results of previous experiments that demonstrated an association between engagement and reduced movement often concurrently showed lower levels of engagement in association with lower levels of user interaction (van den Hoogen et al., 2008). Cognitive engagement was thus not necessarily the sole cause of reduced movement, as physical engagement, and interaction rate influenced the results. Particularly, for games or tutorial systems where interaction rate was controlled by the end user (e.g., exploration games or card games), it would not be possible to determine whether the higher interest caused lower movement rates, or if temporary breaks in gaze and attention (due to pauses in interaction) allowed for more movement.

### Methods

Study 2 was designed as a single independent variable with three levels, each being a different stimulus: two specially constructed, interactive reading stimuli, and a game (see Stimuli Section). This study was conducted with the same participants (and in the same hour-long session) as study 1, using identical instruments and scales as in study 1, as well as identical data analysis and movement measurements, and an identical procedure. Because study 2 included a commercial game (Zuma, see Stimuli Section), participants who had never played Zuma before were instructed in how to play, and allowed to play for 3 min before any measurements were made, in order to prepare them for the experimental playing session later, and to familiarize them with the use of the handheld trackball.

#### Stimuli

In this study, three time-sensitive, interactive stimuli were used: a commercial video game called Zuma (stimulus abbreviation ZU, in which the player has to shoot colored balls at other rolling balls that match its color before the rolling balls reach the finish line), and two reading comprehension tests made in Macromedia Flash Professional 8. These interactive stimuli are summarized in Table 7, and individual frames from these stimuli are shown in Supplementary Figure 3. The reading excerpt that we designed to be interesting came from a best-selling novel, The Curious Incident of the Dog in the Night-time (stimulus abbreviation CIDN; Haddon, 2003), and the boring reading came from European banking regulation (EUB; European Banking Authority, 2013). The reading comprehension tests involved 3 min of reading (~700 words), followed by a single question testing the user's comprehension. The body movement analysis only considered events during the reading, and it excluded the activity associated with the quiz *per se*. To make the reading continuous and time-sensitive, the text crawled up the screen (like movie credits) at a rate of 24.4 lines per min (50 characters per line, approximately allowing four words per second); this meant that looking away from the screen could result in failure.

**Table 7:** **Summary of interactive stimuli used in this experiment**.

**Stimulus**	**Actions/minute**	**Expected design goal**
**READING COMPREHENSION TESTS**		
EU regulations: EUB	27	Boring
Best seller: CIDN	27	Engaging
**COMMERCIAL GAME**		
Game: ZUMA	30–60	Highly engaging

Actions/minute = the expected number of interactions (i.e., trackball clicks) per min. In the reading comprehension tests, the actions occur on a set schedule (the same for both stimuli). Expected design goal refers to the a priori subjective response the research team wanted to elicit from the participants. EUB refers to a reading stimulus based on European Union banking regulations. CIDN refers to a reading stimulus based on “The Curious Incident of the Dog in the Night-time.”

To vouchsafe that the reading tasks had a constant amount of interaction, approximately every 2 s (at inconsistent intervals) the reading was replaced with a gray screen, which remained in place until the user clicked anywhere on the screen with the handheld trackball, after which the reading returned. Volunteers were instructed to click as quickly as possible when they saw the gray screen, as otherwise, they might miss some of the text. This appearing and disappearing feature was described by many participants as slightly irritating, as it kept them on edge during the reading task. This feature meant that the interaction rate for the reading tasks was comparable to the interaction rate of Zuma.

### Results: Interactive stimuli

#### Subjective responses

Subjective ratings for the reading comprehension tests (Descriptive statistics see Table 8) showed that, as expected, the text from the best-selling novel (CIDN) was more engaging than the European banking regulation text (EUB), and the commercial video game was more engaging than the best-selling novel reading [ANOVA, *p* < 0.001, *F*(2, 80) = 76.36, ${\hat{\omega }}^{2}$ = 0.73, *post-hoc* Tukey comparison, see *post-hoc* comparisons Table 9]. The lower engagement ratings of CIDN compared to ZU could be due to the fact that the reading comprehension tests required the participant to interact with the gray-screens (see Methods). The VAS boring ratings for these three tasks suggested that the European banking regulation reading task (EUB) was genuinely boring for everyone who experienced it (VAS boring mean 67.33, s.d. 31.28, IQR 45-95), which is a success, given that some previous attempts to make meaningless, boring tasks/games with high interaction rates have nevertheless generated interest in users who made boring tasks autotelic, through competing with themselves against the clock (Jennett et al., 2008; see also Witchel and Westling, 2013). EUB is significantly more boring than either of the other two interactive tasks [ANOVA, *p* < 0.001, *F*(2, 80) = 40.35, ${\hat{\omega }}^{2}$ = 0.59, *post-hoc* Tukey comparison, *p* < 0.001 for both], while the interesting tasks were not rated significantly differently for boredom (*P* = 0.89). For frustration ratings, the stimuli were all significantly different in the order (starting from most frustrating): banking regulation text (EUB) > best-selling novel (CIDN) > Zuma Game [see Table 8, ANOVA, *p* = 0.001, *F*(2, 80) = 21.06, ${\hat{\omega }}^{2}$ = 0.42, *post-hoc* Tukey comparison, see comparisons Table 9].

**Table 8:** **Subjective ratings elicited by each interactive stimulus**.

	**Study 2 (N = 27)**	
	**Engaging *M* (*SD*)**	**Frustrated *M* (*SD*)**
**READING COMPREHENSION TESTS**		
EU regulations: EUB	23.93 (19.38)	69.44 (30.73)
Best seller: CIDN	61.67 (23.41)	46.30 (34.52)
**COMMERCIAL GAME**		
Game: ZUMA	77.41 (17.23)	22.78 (23.30)

Ratings were made immediately after the stimulus on a 0–100 Visual Analog Scale. Engaging, “I felt totally engaged by the experience.” Frustrated, “I felt frustrated by the experience.” EUB refers to a stimulus based upon reading European Union banking regulations. CIDN refers to a reading stimulus based on “The Curious Incident of the Dog in the Night-time.”

**Table 9:** **Comparison of subjective responses elicited by each interactive stimulus**.

**Stimulus 1**	**Stimulus 2**	**95% CI Lower bound**	**Difference of Means (1–2)**	**95% CI Upper bound**	***P***
**TOTALLY ENGAGED (***N* = 27**)**					
EU Regs: EUB	Best Seller: CIDN	–48.47	–37.74	–27.01	<0.001
Game: ZUMA	EU Regs: EUB	42.75	53.48	64.21	<0.001
Game: ZUMA	Best Seller: CIDN	5.01	15.74	26.47	0.0024
**FRUSTRATED (***N* = 27**)**					
EU Regs: EUB	Best Seller: CIDN	5.80	23.15	40.50	0.0062
Game: ZUMA	EU Regs: EUB	–64.02	–46.67	–29.32	<0.001
Game: ZUMA	Best Seller: CIDN	–40.87	–23.52	–6.17	0.0053

*These are post hoc statistical comparisons (Tukey) of a repeated measures ANOVA. If the difference of means (1–2) is positive, then the mean Visual Analog Scale subjective response elicited by stimulus 1 was higher than stimulus 2, while stimulus 2 elicited higher mean subjective rating score than stimulus 1 if the difference is negative. EUB, a stimulus based on European Union banking regulations. CIDN refers to a reading stimulus based on “The Curious Incident of the Dog in the Night-time.” See Methods for details*.

#### Head movements

In terms of movement elicited, the three interactive stimuli (best-seller CIDN, EU regulations EUB and game ZU) differed highly significantly in terms of elicited head movement [see Table 10, Repeated Measures ANOVA, *p* = 0.017, *F*(2, 80) = 4.42, ${\hat{\omega }}^{2}$ = 0.11, *post-hoc* Tukey comparison, see *post hoc* comparisons Table 11].

**Table 10:** **Mean speed of movement elicited by each interactive stimulus**.

	**Study 2 (N = 27)**	
	**Forehead (mm/s) *M* (*SD*)**	**Thigh (mm/s) *M* (*SD*)**
**READING COMPREHENSION TESTS**		
EU regulations: EUB	0.41 (0.41)	0.12 (0.11)
Best seller: CIDN	0.24 (0.25)	0.07 (0.07)
**COMMERCIAL GAME**		
Game: ZUMA	0.30 (0.19)	0.07 (0.05)

Speed is the absolute value of the Euclidean distance traveled in two-dimensions of a motion capture reflective marker between two time samples (25 Hz); mean speed refers to the average speed over the course of 82 s (see Methods for details). EUB refers to a reading stimulus based upon reading European Union banking regulations. CIDN refers to a reading stimulus based on “The Curious Incident of the Dog in the Night-time.”

**Table 11:** **Comparison of speeds of movements elicited by each interactive stimulus**.

**Stimulus 1**	**Stimulus 2**	**95% CI Lower bound**	**Difference of Means (1–2)**	**95% CI Upper bound**	***P***
**FOREHEAD SPEED (mm/s**, *N* = 27**)**					
EU regs: EUB	Best seller: CIDN	0.03	0.17	0.32	0.0140
Game: ZUMA	EU regs: EUB	–0.26	–0.11	0.03	0.1389
Game: ZUMA	Best seller: CIDN	–0.08	0.06	0.20	0.5889
**THIGH SPEED (mm/s**, *N* = 27**)**					
EU regs: EUB	Best seller: CIDN	0.01	0.05	0.09	0.0148
Game: ZUMA	EU regs: EUB	–0.10	–0.05	–0.01	0.0128
Game: ZUMA	Best seller: CIDN	–0.04	0.00	0.04	0.9983

*These are post-hoc statistical comparisons (Tukey) of a repeated measures ANOVA. If the difference of means (1–2) is positive, then the mean motion elicited by stimulus 1 was higher than stimulus 2, while stimulus 2 elicited more movement if the difference is negative. EUB, a reading stimulus based on European Union banking regulations. CIDN refers to a reading stimulus based on “The Curious Incident of the Dog in the Night-time.” See Methods for details*.

The interactive reading comprehension quizzes elicited significantly different head movement speeds from each other; the mean forehead speed for the boring EU reading test (EUB) was 72% faster than the engaging best-seller (CIDN) reading test (see Table 10 and *post hoc* comparison Table 11). Because these stimuli are highly matched (i.e., they have identically high interaction rates and identical reading rates), this result is a clear example where a cognitive state alone (CIDN's increased cognitive engagement or decreased frustration) can lead to NIMI.

However, increased engagement (or decreased frustration) does not necessarily lead to significant NIMI (i.e., lower head movement speeds); the most engaging interactive stimulus (the commercial game ZU) did not elicit significantly different head movement than either of the reading tests (see Tables 10, 11). The head movement speed elicited by ZU was between the speeds for EUB and CIDN, possibly reflecting the fact that it was highly engaging (thus minimizing non-instrumental movements) but also required instrumental movements of the head to aim the ball at different parts of the screen (increasing total movements). Thus, although ZU was the most engaging and least frustrating interactive stimulus, the mean head speed during the game ZU (0.30 mm/s, s.d. 0.19) was higher than the mean head speed during CIDN (0.24 mm/s, s.d. 0.25), despite the fact that CIDN is less engaging than ZU. This highlights the importance of precisely matching stimuli when determining the cognitive effects of longer stimuli on head movement, and it also reinforces the relevance of stimulus factors other than elicited cognitive states when interpreting the meaning of movements during screen engagement.

#### Thigh movements

The elicited thigh movements differed significantly between the boring stimulus (EUB) and the interesting ones (ZU and CIDN), but not between the two interesting stimuli [see Table 10, Repeated Measures ANOVA, *p* = 0.006, *F*(2, 77) = 5.74, ${\hat{\omega }}^{2}$ = 0.15, *post-hoc* Tukey comparison, see *post-hoc* comparisons Table 11]. Thus, unlike head movement activity, which for an interesting game like ZU is half-way between the boring reading test (EUB) and the interesting one (CIDN), thigh movement for the interesting game (ZU) is virtually identical to that for the interesting reading test (CIDN). We suggest that instrumental head movements driven by shifting gaze (i.e., the instrumental movements required for visual aiming of ZU) do not bleed into the signal from the thigh; instead, we conclude that the thigh's movement is inhibited (i.e., it manifests NIMI) by a person's experience of cognitive engagement alone (i.e., independent of physical engagement with the screen).

#### Head position: Distance from monitor

In terms of mean head distance from the screen (i.e., position, rather than movement), there was a significant difference between the engaging game ZU and the interesting reading test (CIDN), but not between any other pairs of interactive stimuli [Table 12, Repeated Measures ANOVA, *p* = 0.030, *F*(2, 80) = 3.77, ${\hat{\omega }}^{2}$ = 0.09, *post-hoc* Tukey comparison]. The head marker is closer (averaged over time) to the screen (by 10 mm, but not significantly) during the interesting reading test (CIDN, 71.80 cm, s.d. 7.31) than during the boring reading test (EUB, 72.80 cm, s.d. 9.00). However, the most engaging stimulus (Game, ZU) elicits a mean head position that is further away than both reading tests (7 mm average further than EUB, mean distance 73.52 cm, s.d. 8.77). These data do not support the claim that interesting stimuli draw the head closer to the screen; instead, it appears that idiosyncratic or unidentified features of the stimulus control the mean distance of the head from the screen.

**Table 12:** **Comparison of mean distance to the screen during each interactive stimulus**.

**Stimulus 1**	**Stimulus 2**	**95% CI Lower bound**	**Difference of Means (1–2)**	**95% CI Upper bound**	***P***
**FOREHEAD DISTANCE FROM SCREEN (Mean, cm**, *N* = 27**)**					
EU regs: EUB	Best seller: CIDN	–0.51	1.00	2.52	0.2555
Game: ZUMA	EU regs: EUB	–0.80	0.71	2.23	0.4970
Game: ZUMA	Best seller: CIDN	0.20	1.72	3.23	0.0229

*These are measurements of calibrated horizontal distance from the motion capture reflective marker at the forehead to the computer monitor, as measured during 82 consecutive sec of the stimulus; larger numbers imply the head was located further away from the screen. These are post-hoc statistical comparisons (Tukey) of a repeated measures ANOVA. If the difference of means (1–2) is positive, then the mean distance during stimulus 1 was higher (i.e., farther) than during stimulus 2, while stimulus 2 was on average closer if the difference is negative. EUB, a reading stimulus based on European Union banking regulations. CIDN, a reading stimulus based on “The Curious Incident of the Dog in the Night-time.” See Methods for details*.

### Discussion: Interactive stimuli

#### Total movement vs. non-instrumental movement: Targeting gaze

The hypothesis tested in this study was that seated participants decrease their movements in response to more engaging interactive video experiences. EUB and CIDN have precisely identical interaction rates, while ZU has a comparable interaction rate. ZU was included to demonstrate potential exceptions to the rule due to the differences between total movements vs. non-instrumental movements.

The tested hypothesis was strongly supported by the matched reading comprehension quizzes (nearly a two-fold difference in head and thigh movement, *P* < 0.001), but not for ZU. Thus, this data supports our conclusion that cognitive engagement leads to NIMI. However, if all other stimulus factors are not equal, cognitive engagement is not sufficient to lead to decreased total head movement, especially if there may be instrumental head movements to look at different parts of the screen.

#### Head movement vs. thigh movement

In our speed measurements of total head movement, engaging ZU's elicited instrumental head movement speed approaches boring EUB's non-instrumental head movement speed. By contrast, the mean thigh movement levels of ZU are nearly the same as the engaging CIDN. During seated HCI there is rarely an instrumental reason for the participant to move the thigh. This is why measurements of total thigh movements may reveal a difference between engaging and boring stimuli. It should be noted that the thigh makes much less movement than the head, and often it does not move at all during the course of 82 s.

#### Measuring movement in other parts of the body

In this study we chose not to include our shoulder measurements because the head and shoulders reflect similar (but not identical) movement—in particular, whenever the shoulders make movements forward or backward, the head usually moves the same way because the neck's base is connected to the shoulders. We did not measure foot (or hand) movement for several technical reasons. The fact, that the arm and leg can rotate (e.g., supination and pronation) means that for our camera-based set up there will be problems with occlusion of the markers, which would create discontinuous data. This occlusion problem is worsened by furniture. The potential solution is to use wearable inertial sensors, which will provide clear indicators of movement, and under good conditions relatively precise readings of position.

#### Measuring features other than speed and position

Speed (rate of change) is a metric that has emerged in our lab and others (D'Mello et al., 2012; Witchel et al., 2013a,b, 2014a), which has been found to be sensitive to engagement/boredom, and it is the most simple metric for calculating movement (as opposed to position). Our laboratory has linked other movement features to cognitive engagement, including range (which has obvious problems with statistics including not being comparable for different time durations) and the 2-s-window standard deviation of ranges (SD Ranges—SDR, a measure of the variability of variability; Witchel et al., 2014b).

There are many possible features, including acceleration. jerk, standard deviation, kurtosis, skew, entropy, and spectral features (comparing different ranges outputted from Fourier transforms) including spectral energy and the amount of white vs. pink noise (D'Mello et al., 2012). A key aspect for future analysis using many of these alternative features is that they consider the structure of movement (i.e., clustering) rather than the total amount of movement, which does not differentiate small consistent movement from occasional jolts.

## Conclusions

In this paper two studies both involved the use of three stimuli to investigate the claimed hypothesis that video engagement can be recognized by diminished postural movement and that boredom and frustration are associated with more movement. There is currently no way to distinguish instrumental from non-instrumental movements based on the movement records alone. Our approach was, therefore, to design stimuli and interactions that minimized instrumental movements (i.e., by using a handheld trackball instead of a mouse), so that the only instrumental movements were head movements related to the targeting of gaze (and very small finger movements associated with the trackball). We found evidence to support three major conclusions:

Conclusion (1) The primary hypothesis is supported. To the best of our knowledge, study 2 is the clearest example showing that when stimuli are directly comparable (e.g., matched interactivity rates), cognitive engagement is associated with an inhibition of non-instrumental movements.
Conclusion (2) Head movements associated with the targeting of gaze can make a profound difference to the movement results that one detects, and that apparent exceptions to the primary hypothesis can be found if one does not consider instrumental head movements associated with the targeting of gaze. That is, inhibition of head movement is more strongly driven by the need to watch the screen than by cognitive engagement.
Conclusion (3) As a corollary to the above findings, we presented evidence that when people are seated, thigh movement seems to be inhibited during engagement. Thus, NIMI can affect parts of the body that are not necessarily instrumental in gaze targeting. Therefore, NIMI is not just an epiphenomenon of visual attention—it relates to cognitive engagement *per se*.

The novel additions of this study to the literature are: (A) the two reading comprehension stimuli in study 2 (EUB and CIDN) are absolutely comparable; they are the same stimulus except that the words are different, and this difference in words is enough to change both how interesting the visual stimulus is, and how much movement it elicits. (B) The two reading comprehension stimuli in study 2 are highly interactive (27 mouse clicks per min); this means that during the boring stimulus (EUB) the participants were looking at the stimulus, countering the trivial explanation that they were looking around the room rather than looking at the screen. (C) The trivial explanation (if you do not have to look at the screen, you can move your head more) is clearly demonstrated in study 1, and we show that looking around the room elicits much more head movement (mean speed > 0.88 mm/s) than even the most boring stimulus that requires consistent visual attention (EUB mean speed = 0.41 mm/s); the interesting visual stimuli elicited even lower head speeds.

Thus, study 1 shows that increased cognitive engagement is neither necessary nor sufficient to diminish total movement. There are other factors that diminish movement including targeting gaze and attention, increased mouse/keyboard interactions (or other instrumental actions that lock the shoulder in place), and lethargic boredom. The likely factors that increase movement will be high arousal, break-taking, frustration and suppressed escape, emotional expression, and instrumental activity. Furthermore, the development of the universally boring reading comprehension test (EUB) demonstrates that it is possible to have a high interaction rate while being subjectively boring and not engaging; thus, interactivity is not synonymous with cognitive engagement. We theoretically synthesize the conflicting observations from the two literatures mentioned in the introduction (i.e., in museums and standing game-play more movement implies engagement while in HCI more movement implies boredom or frustration) as follows: (1) physical engagement alters instrumental movement, (2) physical engagement tends to cause cognitive engagement, and (3) purely cognitive engagement with fixed screens tends to cause NIMI. Key to progress in this field will be the ability to computationally distinguish instrumental from non-instrumental movements; this may occur with careful analysis of the structure of movement (D'Mello et al., 2012; Witchel et al., 2014b), rather than by simply analyzing its average speed.

Finally, the link between sitting forward and cognitive engagement continues to defy explanation. This study provides two more examples failing to confirm that engagement leads to a forward head position *on average*. While studies on the interpretation of nonverbal behavior consistently make this link (Coan and Gottman, 2007; Sanghvi et al., 2011), carefully measured studies on encoding nonverbal behavior do not consistently do so. Given that the intuitive meaning of leaning forward is both physical and cognitive engagement, there may be something that we are failing to measure, or to take account of, in our measurements.

## Author contributions

HW oversaw the experiments, designed the experiments, and drafted the first manuscript. CS performed most of the experiments, and contributed to the manuscript's completion. JA conceived of the original idea for study 1, and contributed to the manuscript. CW conceived of the original idea for study 2 and its stimuli. She made major corrections to the manuscript, and made several figures plus the video. NC oversaw the technical side of the measurements, including the original work with video tracking.

## Funding

This research was partially funded by a Wellcome Trust Biomedical Vacation Scholarship to CS (105298/Z/14/Z), and in part by the Independent Research Project program run by BSMS to JA and HW.

### Conflict of interest statement

The authors declare that the research was conducted in the absence of any commercial or financial relationships that could be construed as a potential conflict of interest. The reviewer, SG, and handling Editor declared their shared affiliation, and the handling Editor states that the process nevertheless met the standards of a fair and objective review.
