# Supplementary material for: Non-Instrumental Movement Inhibition (NIMI) Differentially Suppresses Head and Thigh Movements during Screenic Engagement: Dependence on Interaction
Source: Front Psychol. 2016 Feb 23;7:157. doi: 10.3389/fpsyg.2016.00157 (PMC4762992; doi:10.3389/fpsyg.2016.00157)
Supplement: Supplementary file 2 [file Witchel_Supplementary_Data_Results.pdf]

Supplementary Data and Results

Non-Instrumental Movement Inhibition (NIMI) differentially suppresses head and thigh movements during screenic engagement: dependence on interaction

Authors and Affiliations

Harry J. Witchel [1,*], Carlos P. Santos [1], James K. Ackah [1], Carina E.I. Westling [2], Nachiappan Chockalingam [3]

[1] Brighton and Sussex Medical School (BSMS), University of Sussex, Brighton BN1 9PX, UK
[2] School of Media Film and Music, University of Sussex, Brighton, UK
[3] CSHER, Staffordshire University, Stoke-on-Trent, UK

For
Frontiers in Psychology (2016, Feb). Vol 7: Article 157. doi: 10.3389/fpsyg.2016.00157

Keywords: posture, movement, human, cognitive engagement, NIMI, motion capture, video tracking, Non-Instrumental Movement Inhibition.

Table of Contents

Supplementary Tables 2
Supplementary Table 01. Paired OK Go musical stimuli that different participants experienced..2
Supplementary Figures 3
Supplementary Figure 01. Sample images from the musical stimuli in study 1..................3
Supplementary Figure 02. Movement traces in response to stimuli. 4
Supplementary Figure 03. Sample images from interactive stimuli in study 2 6
Supplementary Videos 8
Supplementary Video 01. Engaging music elicits dancing, boredom elicits fidgeting. 8

Supplementary Tables

Supplementary Table 01. Paired OK Go musical stimuli that different participants experienced.

Study 1 used a total of five stimuli, four of which are OK Go music video stimuli (see Table 01 in the main paper); however, each participant experienced only three stimuli: one of them was their favorite song (without video) and two of them were matched stimuli based on OK Go music videos (i.e. one of the two rows in the table in panel A). Thus, they never heard the same song twice, in order to avoid habituation and boredom effects. Participants were assigned to stimulus pair I or stimulus pair II in a counterbalanced order. Panel B shows the complete counterbalance corresponding to panel A; each row of this counterbalance had either 2 or 3 participants assigned to it. Note that the cardinal numbering (first, second, third) is relative and not absolute; all participants initially experienced two training stimuli.

Panel A

	Multimodal	***Audio Only***
Stimulus Pair I	Do What You Want (music video)	Here It Goes Again (music with black screen)
Stimulus Pair II	Here It Goes Again (music video)	Do What You Want (music with black screen)

Panel B

Counterbalance #	***Stimulus Pair***	***First Stimulus***	***Second Stimulus***	***Third Stimulus***
1	II	FAV	HIGA multi	DWYW audio
2	II	HIGA multi	FAV	DWYW audio
3	II	HIGA multi	DWYW audio	FAV
4	I	FAV	HIGA audio	DWYW multi
5	I	HIGA audio	FAV	DWYW multi
6	I	HIGA audio	DWYW multi	FAV
7	II	FAV	DWYW audio	HIGA multi
8	II	DWYW audio	FAV	HIGA multi
9	II	DWYW audio	HIGA multi	FAV
10	I	FAV	DWYW multi	HIGA audio
11	I	DWYW multi	FAV	HIGA audio
12	I	DWYW multi	HIGA audio	FAV

Supplementary Figures

Supplementary Figure 01. Sample images from the musical stimuli in study 1.

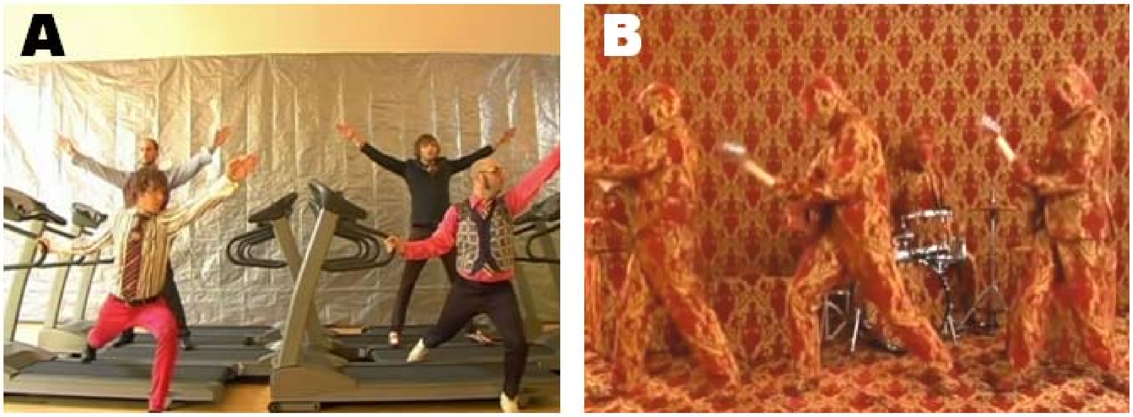

Panel A shows a frame from the music video by the band OK Go for the song “Here It Goes Again”, which was danced on treadmills (https://youtu.be/dTAAsCNK7RA). Panel B shows a frame from the music video “Do What You Want” (version 2 Wallpaper) by OK Go (https://youtu.be/i00GDT9FuFM). In this video, a peculiar red and yellow flocked wallpaper forms both the backdrop and the outfits for the band, dancers and acrobats who appear in the video; as can be seen, no faces are visible because all people in the film wore masks made of the same wallpaper pattern. Still frames from videos included as fair use for research; all copyrights EMI Music.

Supplementary Figure 02. Movement traces in response to stimuli.

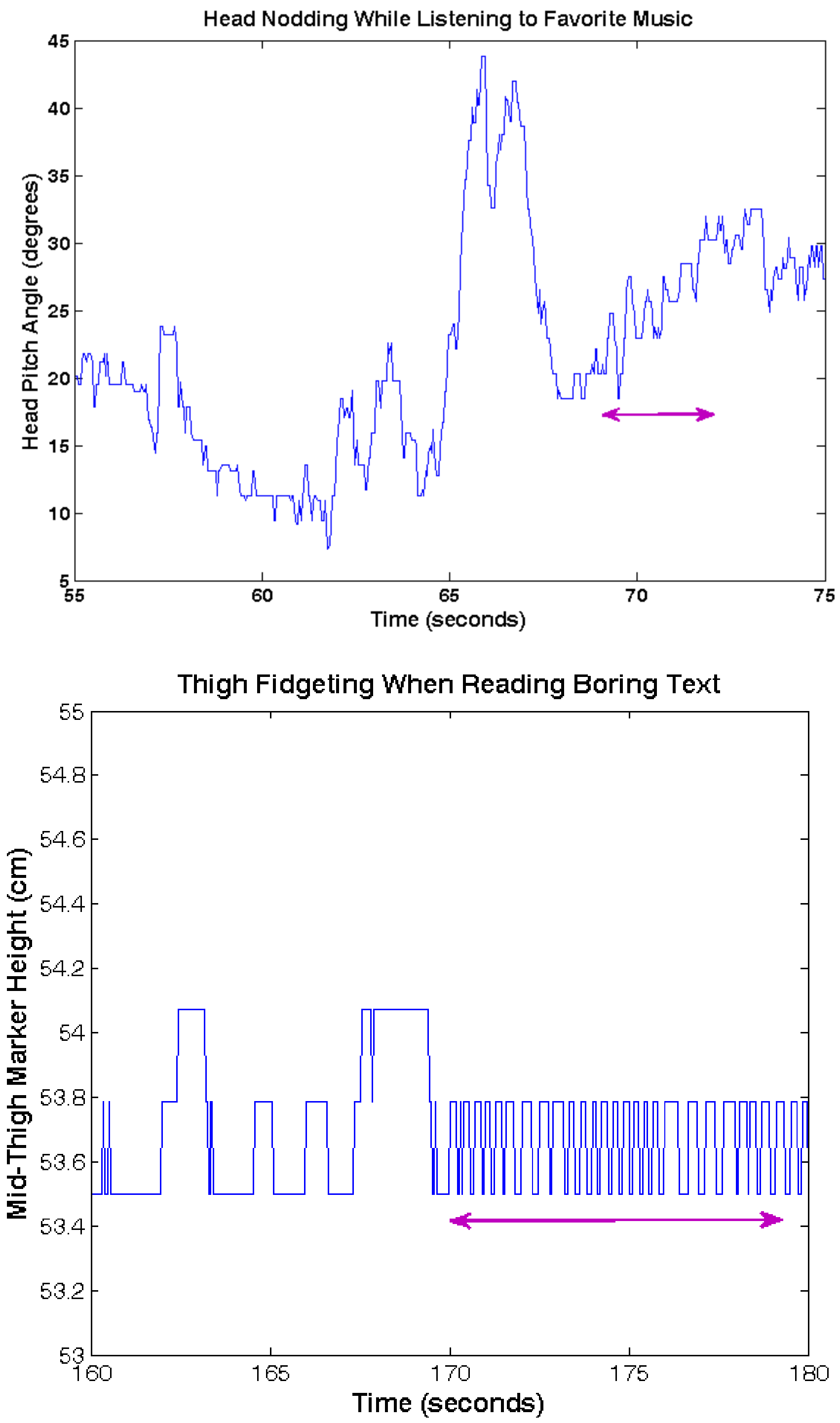

Supplementary Figure 02. Raw movement traces in response to stimuli

This data accompanies Supplementary Video 01 in order to demonstrate non-instrumental fidgeting and rhythmic movements to music

The upper panel shows a raw movement trace of head angle vs. time (blue) for the participant shown in segment 1 (Y065) of supplementary video 01 as she was listening to

her favorite music (FAV). The double-headed arrow (purple) shows a five second period (near the end of the filmed segment) when she was nodding rhythmically in time with the music (one head nod per half second). The head angle refers to the angle deviating from the horizontal line (i.e. the floor) made by a line running between the marker on her forehead (outer canthus of the eye) and the marker on the pinna of her ear. The lower panel shows a raw movement trace of mid-thigh height (from the floor, in cm) vs. time (blue) for the participant shown in segment 2 (Y090) of supplementary video 01 as she was reading a boring text taken from European Union Banking regulations (EUB). The double-headed arrow (purple) shows the ten-second segment of the film, while the rest of the trace (before time 170 sec) shows the previous dearth of fidgeting movements for context. The jaggedness of the trace shows the limits of resolution of this video tracking procedure with these films, which has an absolute limit of ~ 1 mm. While these thigh movements are quite small (a few millimeters), the movements of the fibula marker during this kind of fidgeting were larger (~2 cm).

Supplementary Figure 03. Sample images from interactive stimuli in study 2

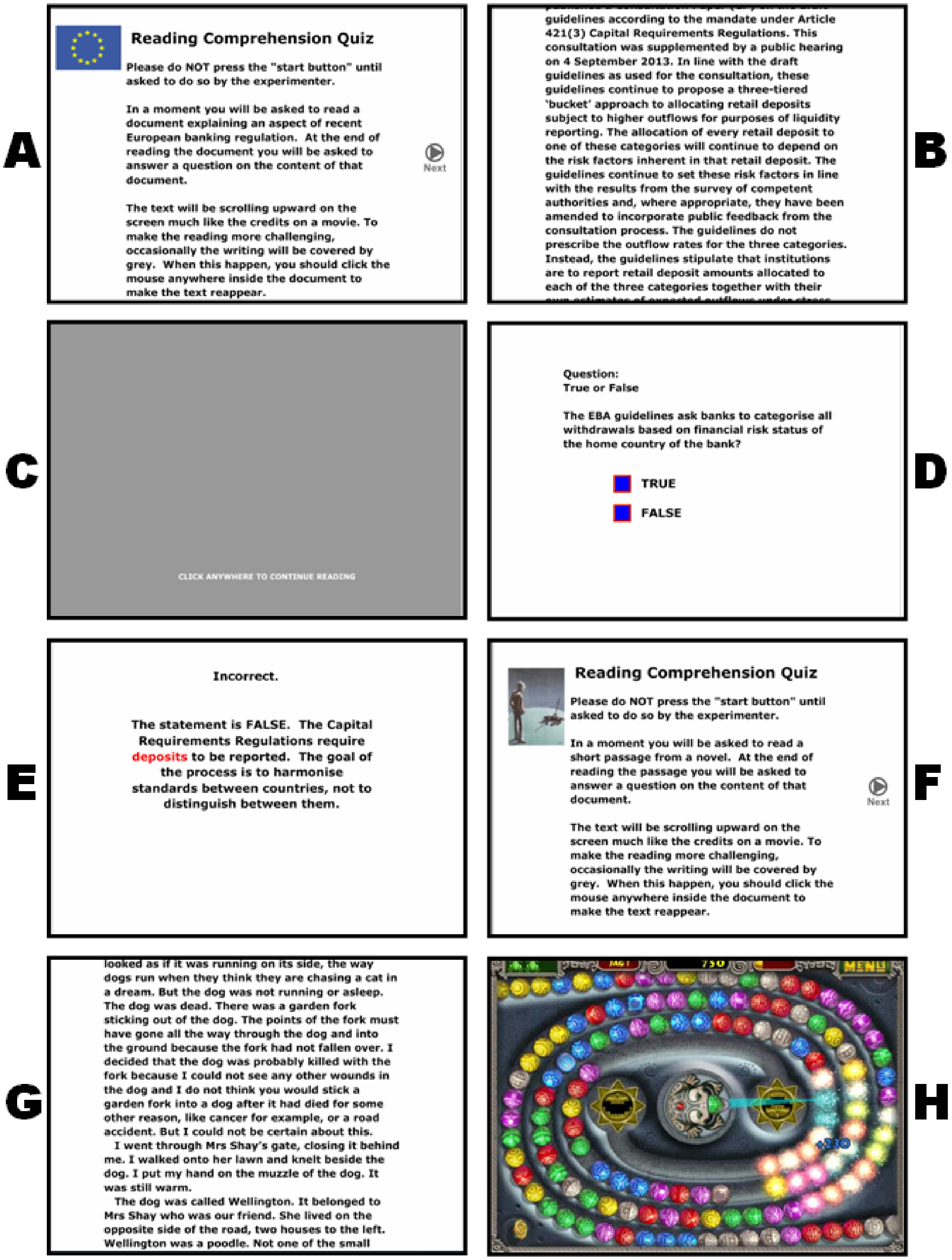

Supplementary Figure 03. Sample images from interactive stimuli in study 2
Panels A-E show sample images from the European Union Banking Regulation reading comprehension quiz (EUB), which was made in Flash. Panel A shows the instructions screen; this screen remains in place until the "next" arrow button at the right is pressed by the

participant after the experimenters leave the room. Panel B shows a sample from the text in EUB; note that the top and bottom are cut off because the text is slowly crawling up the screen as in movie credits. During the quiz, the Flash window was expanded to full screen size, so in the screen visible to the participant, which was 47.5 x 27 cm, the paragraph text font (Verdana 14 point bold in Flash's design mode) achieved a height of 6 mm tall for capital letters and 4.5 mm tall for lowercase letters; this size text can be easily read from a distance of 4 meters by a person with normal vision. Our participants were free to adjust the chair position to a comfortable distance, and most participants positioned themselves such that their eyes were 70-80 cm from the monitor, so the letters subtended a viewing angle of approximately 0.43 degrees for capital letters and 0.37 degrees for lowercase. Panel C shows the grey screen that blocks the reading; this screen appears approximately every two seconds. The white writing on the grey screen is, "Click anywhere to continue reading". Panel D shows the question at the end of this quiz. Panel E shows the feedback screen that appears after the participant answers the question in panel D (in this case the participant clicked on "TRUE", which is the wrong answer). Note that the movements of the participant during panels D and E are not analyzed (because the primary activity is not reading), although the participant is not told this. Panel F shows the instruction screen for the reading comprehension test for the Curious Incident of the Dog in the Night-time (CIDN). The screen and instructions are nearly identical to panel A. Panel G shows some sample text from CIDN, and this text would be slowly crawling up the screen (reprinted with permission, copyright Random House, all rights reserved). Panel H shows a sample screen from the game ZUMA by PopCap games (with general permission, all rights reserved by Electronic Arts). In this game, the participant must rapidly shoot colored balls from the frog's mouth (center of screen) at the surrounding balls to create sets of three or more adjacent balls with the same color.

Supplementary Videos

Supplementary Video 01. Engaging music elicits dancing, boredom elicits fidgeting.

This video is located on the Frontiers in Psychology website:

http://journal.frontiersin.org/article/10.3389/fpsyg.2016.00157

Two anonymised participants are shown making small movements in response to the stimuli they experience. In segment 1 of the video, a participant (Y065) is listening to her favorite music. The elicited actions that occur include singing along (not visible), jigging her leg in time to the rhythm, nodding her head in time to the rhythm (near the end of this segment), and shifting her torso/shoulders once. None of the actions are persistent or continuous, nor was the participant given instructions to interact with the music. In segment 2 of the video, a participant (Y090) is interacting with a very boring stimulus (reading a text from European Union Banking Regulation while having to click on a trackball repeatedly). The elicited fidgeting and other non-instrumental actions that occur include rapidly and rhythmically jigging both legs, pitching her head forward and back, and talking to the computer monitor (not visible, she says, "Oh come on!"). These two examples show that over the course of only one minute, seated people sometimes make a fair amount of movement a) as non-instrumental emotional responses to what is occurring, and b) as non-instrumental postural activities (i.e., for no reason at all except that they feel their bodies need to move). This non-instrumental movement occurs, notwithstanding the fact that the participants may feel self-conscious, they can see that they are being filmed, and they know that they are taking part in a psychology experiment. Because people naturally move so much, we propose that the diminution (or absence) of some movements (i.e., what does not happen) can be as revealing as what does occur.
